# Impacts of replacing soybean meal with processed soybean meal on intestinal health and growth of nursery pigs challenged with F18+ *Escherichia coli*

**DOI:** 10.5713/ab.24.0566

**Published:** 2024-10-28

**Authors:** Zixiao Deng, Hyunjun Choi, Sung Woo Kim

**Affiliations:** 1 Department of Animal Science, North Carolina State University, Raleigh, NC 27695, USA

**Keywords:** F18+ *Escherichia coli*, Intestinal Health, Nursery Pigs, Soybean Meal

## Abstract

**Objective:**

This study aimed to investigate the impact of different level of soybean meal (SBM) replaced by soy protein concentrate on intestinal health and growth performance of nursery pigs under F18+ *Escherichia coli* (*E. coli*).

**Methods:**

Forty-eight newly weaned pigs (6.6±0.3 kg) were randomly allotted to 4 treatments arranged by 2×2 factors using randomized complete block design with initial body weight and sex as blocks. Two factors were F18+ *E. coli* challenge (0 or 2.1×10^10^ colony-forming units [CFU]) and the level of SBM (24% or 12% in phase 1 and 26% or 14% in phase 2). Pigs were fed for 25 d in 2 phases (phase 1 for 11 d and phase 2 for 14 d). At the end of study, all pigs were euthanized to collect jejunal mucosa and tissues.

**Results:**

The F18+ *E. coli* challenge decreased (p<0.05) overall average daily gain (ADG) and average daily feed intake (ADFI) and decreased (p<0.05) gain to feed ratio on d 7 to 11. The high SBM tended to have a greater overall ADG (p = 0.054) and ADFI (p = 0.078) compared with low SBM under F18+ *E. coli* challenge, but not in unchallenged conditions. The F18+ *E. coli* challenge increased (p<0.05) fecal score on d 7 to 18. The tumor necrosis factor-α and interleukin-1β in jejunal mucosa were decreased (p<0.05) in high SBM treatments. The high SBM tended to increase (p = 0.085) occludin expression in jejunum. high SBM increased crypt depth in jejunum under F18+ *E. coli* challenge, but not in unchallenged conditions (p<0.05).

**Conclusion:**

High SBM in nursery diets could alleviate the detrimental effects of F18+ *E. coli* challenge on growth performance of pigs under compared to low SBM inclusion, which might be attributed to decreased intestinal inflammation and improved intestinal integrity.

## INTRODUCTION

Soybean meal (SBM) is a common protein supplement in pig diets due to its highly digestible and balanced amino acids and functional compounds. However, anti-nutritional compounds such as allergenic proteins, trypsin inhibitors, lectins, and soluble non-starch polysaccharides in SBM limited its use in nursery diets because of their negative impacts on intestinal health and growth performance [1,2]. Aqueous alcohol washing is one of the common methods to manufacture soy protein concentrate (SPC), which removes soluble carbohydrates, denatures soy allergenic proteins, and improves protein content in the final product [3]. Therefore, SPC has been used to replace SBM in the diets of nursery pigs improving growth performance [4]. The beneficial effects of using SPC on intestinal health and growth of nursery pigs are attributed to the removal of glycinin and β-conglycinin, which could cause hypersensitivity, and the reduction of flatulence producing oligosaccharides such as stachyose, raffinose, and verbascose [5,6]. However, aqueous alcohol washing can also reduce certain functional compounds in SBM, such as isoflavones [7]. Genistein and daidzein are the two major isoflavones in soybeans and they have anti-inflammatory and antioxidative effects [8]. Isoflavones could enhance the expression of antioxidant genes and inhibit the production of pro-inflammatory cytokines and chemokines, such as IL-1β, IL-6, IL-12, and tumor necrosis factor-α (TNF-α) via the NF-κB transcriptional pathway [9,10]. Currently, the majority of studies on replacing SBM with processed SBM have been conducted under unchallenged conditions, as SBM primarily provide amino acids for the growth of pigs. Consequently, the functional compounds in SBM are often overlooked in these studies.

The F18+ *Escherichia coli* (*E. coli*) has been identified as a prevalent strain associated with post-weaning diarrhea (PWD) in nursery pigs worldwide, resulting in a significant risk to their intestinal health and growth performance [11]. Various strategies have been used to alleviate the detrimental effects of pathogen infections, such as antibiotics, probiotics, and prebiotics [12]. Therefore, soy isoflavones have been used in pig diets to attenuate the challenge of lipopolysaccharide and virus infection, such as porcine reproductive and respiratory syndrome virus (PRRSV) [13–15]. Zhu et al [13] indicated that supplementation of 40 mg/kg isoflavones in the diets of nursery pigs could alleviate intestinal barrier damage caused by lipopolysaccharides. In addition, Rochell et al [14] showed that increased SBM inclusion (from 17.5% to 29.0%) in nursery diets could positively modulate immune responses by lowering proinflammatory cytokines such as, haptoglobin and TNF-α, and improve growth performance of pigs under PRRSV infections.

Even though the replacing SBM with processed SBM to reduce allergenic proteins could improve health and growth of nursery pigs under unchallenged conditions [16,17], it is still interesting to investigate the effects of SBM containing high level of isoflavones on the intestinal health and growth of pigs under F18+ *E. coli* challenge, because anti-inflammatory and antioxidative properties of isoflavones may help pigs cope with challenge conditions. Considering the cost of processed SBM, reducing its use under challenge conditions could be a practical way to reduce feed cost. In addition, an investigation about the balance between negative effects of allergenic proteins and positive effects of isoflavones in SBM would provide applicable information to determine the level of SBM and processed SBM in nursery diets for different health status.

Based on the previous findings, it is hypothesized that feeding SBM to nursery pigs challenged with F18+ *E. coli* would benefit intestinal health and growth performance due to functional compounds in SBM compared with feeding processed SBM deprived of these functional compounds. To test this hypothesis, this study evaluated the impact of different levels of SBM replaced by SPC on intestinal health and growth performance of nursery pigs under the F18+ *E. coli* challenge conditions.

## MATERIALS AND METHODS

The procedure of this study was reviewed and approved by North Carolina State University Animal Care and Use Committee, following the North Carolina State University Animal Care and Use Procedures (REG 10.10.01). The experiment was conducted at the North Carolina State University Metabolism Educational Unit (Raleigh, NC).

### Experimental design, animals, and diets

Forty-eight newly weaned pigs (24 males and 24 females; initial body weight (BW) 6.6±0.3 kg) were randomly assigned to four treatment groups arranged by 2×2 factors using a randomized complete block design, with initial body weight (heavy and light) and sex (barrows and gilts) as the blocking factors. Each pig was housed in an individual pen, with 12 replicates per treatment group. Two factors were (1) F18+ *E. coli* challenge (0 or 2.1×10^10^ colony-forming units [CFU]); (2) levels of SBM (24% or 12% in phase 1 and 26% or 14% in phase 2). SPC was manufactured by aqueous alcohol washing. The anti-nutritional compounds and isoflavones contents in SBM and SPC were showed in Table 1. Experimental diets (Table 2) were formulated to meet or slightly exceed the nutrient requirement estimates suggested by NRC [18]. All experimental diets were produced at the Feed Mill Educational Unit at North Carolina State University. The experiment lasted for 25 days and was divided into two phases: phase 1 (weaning to d 11) and phase 2 (d 11 to 25). The duration of each phase was set based on the average BW of pigs as suggested by NRC [18]. Pigs had free access to diets and water during the experiment. The BW of pigs and diets disappearance were measured on d 0, 7, 11, 18, and 25 to calculate average daily gain (ADG), average daily feed intake (ADFI), and gain to feed ratio (G:F).

The F18+ *E. coli* was orally inoculated to 24 pigs (challenged treatments) on d 7 of the study (2.1×10^10^ CFU) of strain F18ac (O147) that was originally isolated from piglets with PWD and produces heat-stable toxins A (STa) and heat-stable toxins B (STb) [19]. Pigs in unchallenged group received a sterile saline solution. The cultures of the F18+ *E. coli* strains F18ac (O147) that produce STa and STb, were prepared following the protocol as previously reported [20]. The fecal score was also recorded during the experiment using a 1 to 5 scale: (1) very firm stool, (2) normal firm stool, (3) moderately loose stool, (4) loose and watery stool, and (5) very watery stool.

### Sample collection and processing

Blood samples (10 mL) were collected from pigs in all pens on d 28 using vacutainer tubes (Becton Dickinson Vacutainer Systems, Franklin Lakes, NJ, USA) by puncturing the jugular vein with 0.8 mm×32 mm needles (Eclipse; Becton Dickinson Vacutainer Systems). The samples were allowed to clot at 4°C for 4 h before being centrifuged at 3,000×g for 15 min (5811F; Eppendorf, Hamburg, Germany). The serum was then aliquoted into 1.5 mL tubes and stored at −80°C until further analysis. Blood sampling was performed 4 d before the experiment concluded to minimize potential stress impacts on the final data.

At the end of the experiment, all pigs were euthanized by exsanguination following the use of a captive bolt. Jejunal tissues and mucosa were then collected. The jejunal tissues were flushed with saline solution to remove digesta, and the mucosa was obtained by scraping the mucosal layer with a glass microscope slide. The jejunal tissues were divided into two pieces: one piece was stored at −80°C, and the other was fixed in 10% buffered formaldehyde. Other tissues and fecal samples were also stored at −80°C until further analysis.

### Oxidative damage products and immune responses in the jejunum

Jejunal mucosa (0.5 g) was weighed and suspended in 1 mL of phosphate-buffered saline (PBS) on ice, then homogenized using a homogenizer (#15340163; Thermo Fisher Scientific, Inc., Waltham, MA, USA). The homogenized sample was transferred to a 2 mL microcentrifuge tube and centrifuged at 14,000×g for 15 min. The supernatant was pipetted into six tubes and stored at −80°C. Concentrations of total protein, protein carbonyl, TNF-α, malondialdehyde (MDA), interleukin 6 (IL-6), IL-8, and immunoglobulin A (IgA) were measured using commercial kits according to the manufacturer’s instructions and Deng et al [21]. The optical density was read with an enzyme-linked immunosorbent assay (ELISA) plate reader (Synergy HT; BioTek Instruments, Winooski, VT, USA) and analyzed using Gen5 Data Analysis Software (BioTek Instruments). Concentrations were calculated based on absorbance from standard curves and the instruction manual. The homogenized mucosal supernatant was diluted 1:30 in PBS to achieve a range of 20 to 2,000 μg/mL, and total protein concentration was measured with the Pierce BCA Protein Assay Kit (#23225, Thermo Fisher Scientific). Absorbance was measured at 562 nm, and total protein concentration was used to normalize other measurements in the mucosa.

The concentration of TNF-α was determined using the Porcine TNF-α Immunoassay Kit (#PTA00; R&D Systems, Minneapolis, MN, USA). Absorbance was measured at 450 nm and corrected at 570 nm, with results expressed in pg/mL of protein. The concentration of IL-6 was measured by using the Porcine IL-6 DuoSet ELISA Kit (#DY686; R&D Systems), with samples diluted 1:5 in reagent diluent. Absorbance readings were taken at 450 nm and corrected at 570 nm, and concentrations were reported as pg/mL of protein. For IL-8, the Porcine IL-8/CXCL8 DuoSet ELISA Kit (#DY535; R&D Systems) was used, following the provided protocol. The mucosal supernatant was diluted 1:5 with reagent diluent, and absorbance was measured at 450 nm and corrected at 570 nm. IL-8 concentrations were expressed as ng/mg of protein. Protein carbonyl was measured using the OxiSelect Protein Carbonyl ELISA Kit (#STA-310; Cell Biolabs, Inc., San Diego, CA, USA). Supernatants were diluted in PBS to achieve a concentration of 10 μg/mL. Standards ranged from 0.375 to 7.5 nmol/mg protein. Absorbance was read at 450 nm, and concentrations were expressed as nmol/mg protein. The concentration of MDA in mucosa was measured by using OxiSelect TBARS MDA Quantitation Assay Kit (#STA-330, Cell Biolabs, Inc.), with a standard working range of 0 to 125 μM. Absorbance was measured at 532 nm, and results were reported as μmol/mg protein. The concentration of immunoglobulin A (IgA) was measured by using the ELISA kits (E101–102; Bethyl Laboratories, Inc., Montgomery, TX, USA). Mucosal supernatants were diluted 1:1000 in PBS before measurement. Absorbance was read at 450 nm, with IgA concentrations expressed as μg/mg of protein.

### RNA extraction and gene expression of tight junction proteins and inflammation

Frozen mid-jejunal tissue (50 to 100 mg) was homogenized in 1 mL of Trizol reagent (#15-596-026; Invitrogen, Waltham, MA, USA) using a Bead Mill 24 homogenizer (#15-340-163; Thermo Fisher Scientific Inc.). The tissue was homogenized twice at 4.5 m/s for 30 s each, with a 20 s rest on ice between sessions. The homogenized samples were then centrifuged at 12,000×g for 10 min at 4°C. The supernatants were transferred to 1.5 mL centrifuge tubes and mixed with 200 μL of chloroform (#146543, Thermo Fisher Scientific Inc.) by gentle shaking for 1 min. The tubes were incubated at room temperature for 10 minutes, followed by centrifugation at 12,000×g for 15 min at 4°C. The aqueous phase was carefully transferred to a new tube and mixed with 200 μL of isopropanol (#B0518327; Acros Organics, Geel, NJ, USA) by gentle shaking for 1 min. After a 10 min incubation at room temperature, the samples were centrifuged at 12,000×g for 15 min at 4°C. The supernatant was removed, and the RNA pellet was washed with 75% ethanol. RNA was eluted with RNase-free water (#2202321, Invitrogen, Austin, TX, USA). The quantity and quality of the eluted RNA were assessed using spectrometry. The extracted RNA was reverse transcribed into cDNA using the RevertAid First Strand cDNA Synthesis Kit (#01299151; Thermo Fisher Scientific Inc.), following the manufacturer’s instructions. Quantitative reverse transcription (RT)-polymerase chain reaction (PCR) (qRT-PCR) was performed with the CFX Connect Real-Time PCR Detection System (BioRad, Hercules, CA, USA) and Maxima SYBR Green/ROX qPCR Master Mix (#01292815; Thermo Fisher Scientific Inc.). Oligonucleotide primers were synthesized by Millipore Sigma (Burlington, MA, USA). Glyceraldehyde-3-phosphate dehydrogenase (GAPDH) was the housekeeping gene. Delta-delta Ct values were calculated to determine relative mRNA levels, with relative expression normalized and expressed as a ratio to the expression in unchallenged low SBM treatment.

### Intestinal morphology and crypt cell proliferation in the jejunum

Mid-jejunal tissues from each pig were analyzed to determine intestinal morphology and crypt cell proliferation. Tissues were fixed in 10% buffered formaldehyde for 48 h. Two sections of the fixed tissue (approximately 2 mm each) were cut, placed in a cassette, and transferred to a 70% ethanol solution. The samples were then sent to the University of North Carolina Histology Laboratory (UNC School of Medicine, Chapel Hill, NC, USA) for dehydration, embedding, and Ki-67 staining. The Biocare Intellipath Stainer (Biocare Medical, Pacheco, CA, USA) was used for automated Ki-67 staining. The primary monoclonal antibody for Ki-67 (#ACR325; Biocare Medical) was diluted 1:100 and incubated with the processed slides for 30 min at room temperature. Vector ImmPress Rabbit polymer was used for detection, and staining was done using chromogen diaminobenzidine (DAB).

Intestinal morphology (villus height [VH], villus width, and crypt depth [CD]) was measured at 40×magnification using an Olympus CX31 microscope (Lumenera Corporation, Ottawa, ON, Canada) and Infinity 2–2 digital CCD software, following the procedure described by Jang et al [22]. Ten complete villi and crypts were chosen to represent the intestinal morphology of each pig. VH was measured from the tip to its junction with the crypt, while villus width was recorded at the midpoint. CD was determined from the base of the villus to the bottom of the crypt. The ratio of villus height to crypt depth (VH:CD) was calculated by dividing the villus height by the CD. The percentage of Ki-67 positive cells, indicating proliferating cells in the crypt, was determined using images of 10 complete crypts captured at 100×magnification with the Olympus CX31 microscope, following the procedure by Xu et al [20]. All morphological analyses were conducted by the same individual person, with the average of 10 measurements per sample reported as a single value.

### Anorectic hormone in blood

Porcine cholecystokinin (CCK) ELISA Kit (MBS264395; Mybiosource, Vancouver, British Columbia, Canada) was used for anorectic hormone measurement. The absorbance was read at 450 nm wavelength and corrected at 630 nm wavelength. The concentration was calculated according to the standard curves and expressed as pg/mL.

### Statistical analysis

Data were analyzed by the Mixed procedure of SAS 9.4 (SAS Institute, Inc., Cary, NC, USA). The initial BW and sex were considered blocking criteria. In the model, the level of SBM (low SBM vs. high SBM) and challenge conditions (F18+ *E. coli* challenged vs. unchallenged conditions) were determined as fixed effects and random effects were sex and initial BW. The least squares of means for each treatment were calculated. Orthogonal contrasts were conducted to test the effect of SBM level, F18+ *E. coli* challenge conditions, and the interaction between the effect of SBM level and F18+ *E. coli* challenge conditions. When an interaction between SBM level and F18+ *E. coli* challenge conditions was significant or tended to be significant, a pairwise comparison was made using the PDIFF option in SAS. Experimental unit was a pen. The p values less than 0.05 were considered statistically significance and between 0.05 and 0.10 were considered tendency.

## RESULTS

### Growth performance and fecal score

The initial BW of nursery pigs was 6.6±0.3 kg in this experiment and there was no difference on BW, ADG, and G:F prior to challenge among treatments (Table 3). The F18+ *E. coli* challenge reduced (p<0.05) BW of nursery pigs on d 11, 18, and 25. The low SBM reduced (p<0.05) BW of nursery pigs on d 11 regardless F18+ *E. coli* challenge. Interaction effects were observed in BW, the low SBM reduced (p<0.05) BW of nursery pigs on d 11, 18, and 25 under challenge conditions. The F18+ *E. coli* challenge reduced (p<0.05) ADG on d 7 to 11, d 11 to 18, d 7 to 25, and d 0 to 25 regardless the level of SBM. The low SBM reduced (p<0.05) ADG on d 7 to 11 regardless F18+ *E. coli* challenge. Under challenge conditions, low SBM reduced (p<0.05) ADG on d 7 to 11 and d 0 to 25. The F18+ *E. coli* challenge decreased (p<0.05) ADFI on d 7 to 11, d 11 to 18, d 18 to 25, d 7 to 25, and d 0 to 25 regardless the level of SBM. The low SBM reduced (p<0.05) ADFI on d 7 to 25 and tended to reduce ADFI on d 18 to 25 (p = 0.088) and d 0 to 25 (p = 0.050). Under challenge conditions, low SBM reduced (p<0.05) ADFI on d 11 to 18 and d 0 to 25. The F18+ *E. coli* challenge reduced (p<0.05) the G:F on d 7 to 11 and tended to increase (p = 0.088) G:F on d 18 to 25. The high SBM tended to have a greater overall ADG (p = 0.054) and ADFI (p = 0.078) compared with low SBM under F18+ *E. coli* challenge, but not in unchallenged conditions. The F18+ *E. coli* challenge increased (p<0.05) fecal score on d 7 to 11, and d 11 to 18 (Table 4). The level of SBM did not affect fecal score regardless F18+ *E. coli* challenge.

### Oxidative damage products, immune responses and gene expression of tight junction proteins and inflammation

The F18+ *E. coli* challenge decreased (p<0.05) IL-8 concentration in jejunum of nursery pigs (Table 5). The high SBM reduced (p<0.05) TNF-α concentration in jejunum. Under challenge conditions, the high SBM decreased (p<0.05) IgA concentration in jejunum.

The F18+ *E. coli* challenge did not affect the gene expression of tight junction proteins. The high SBM tended to increase (p = 0.085) occludin (OC) expression in jejunum of nursery pigs. The F18+ *E. coli* challenge reduced (p<0.05) IL-1β in jejunum. The high SBM reduced (p<0.05) IL-1β in jejunum.

### Intestinal morphology and crypt cell proliferation in the jejunum and anorectic hormone in blood

The F18+ *E. coli* challenge reduced (p<0.05) CD of jejunum of nursery pigs and increased (p<0.05) VH:CD in jejunum (Table 6). The high SBM increased (p<0.05) crypt depth (CD) in jejunum under F18+ *E. coli* challenge, but not in unchallenged conditions. No difference of CCK was observed among treatments.

## DISCUSSION

SPC was commonly used to replace SBM in nursery diets due to the high nutritional values and low anti-nutritional compounds and was capable of partly reducing the use of high-cost animal protein supplements in the diets without negatively affecting growth performance of pigs [4,23,24]. In this study, even though the reduction of SBM replaced by SPC in nursery diets did not show statistical improvement in overall growth performance of pigs, it numerically improved the BW and ADG, which was in agreement with previous study [4]. Due to the epitheliochorial structure of the porcine placenta, newborn piglets must obtain maternal immunoglobulins through ingested colostrum and milk to gain passive immune protection until their own immune system fully matures [25]. This acquired immunity is crucial for defense against pathogens and ensuring survival of piglets. The F18+ *E. coli* challenge impaired the growth performance of nursery pigs, and it might be associated with PWD caused by F18+ *E. coli.* The fecal score of nursery pigs increased after F18+ *E. coli* challenge in all challenged treatments. The enterotoxins Sta and Stb, which are produced by enterotoxigenic *E. coli* (ETEC), including F18+ *E. coli*, play an important role in inducing diarrhea in pigs [11]. This diarrhea is primarily attributed to interference with electrolyte balance due to fimbria receptor interactions. Specifically, the fimbria of *E. coli* binds to glycoproteins that exists on the surface of enterocytes, exacerbating the disruption of the function of enterocytes [26]. The F18+ *E. coli* challenge did not change overall feed efficiency of nursery pigs in this study, therefore, the impaired ADG was mainly attributed to reduced feed intake, which was similar to previous studies [20,27].

The aqueous alcohol washing reduced allergenic protein, trypsin inhibitor, and oligosaccharides and increased protein level in SPC compared to SBM, which was in agreement with a previous report [3]. These anti-nutritional compounds have been indicated to negatively affect intestinal health and growth performance of nursery pigs [1,28]. Interestingly, under F18+ *E. coli* challenge conditions, high inclusion of SBM alleviated the detrimental effects of F18+ *E. coli* challenge compared to low SBM treatment and SPC replacement with lower anti-nutritional compounds did not positively affect the growth performance of nursery pigs. The content of isoflavones in SBM may play a key role in the reduction of growth performance under F18+ *E. coli* challenge. Isoflavones are known for their anti-inflammatory and anti-oxidative properties and interact with various receptors and pathways, including the inhibition of NF-κB activation and inducible nitric oxide synthase enzymes, which are attributed to their antiviral properties [29]. Aqueous alcohol washing could remove isoflavones in the soy flours, and using low levels of SBM in diets of nursery pigs may increase the risk of potential pathogens infections compared to high inclusion of SBM in the diets [30]. Researchers indicated that increasing 11.5% of SBM in nursery diets could positively modulate the immune responses by reducing proinflammatory cytokines under PRRSV infection and then improve the growth performance of pigs [14]. In addition, the supplementation of 40 mg/kg isoflavones in nursery diets could attenuate the negative effects caused by lipopolysaccharides, which might be contributed by improved intestinal integrity [13]. In this study, a 12% reduction of SBM decreased around 200 mg/kg isoflavones in the nursery diets, which could be one of the reasons causing growth impairment of pigs under *E. coli* challenge conditions.

The high SBM decreased TNF-α level in jejunal mucosa compared to low SBM under F18+ *E. coli* conditions. The TNF-α is a pro-inflammatory cytokine that plays an essential role in the synthesis of acute phase proteins and pathogenesis of various infections [31]. In addition, the expression of IL-1β was decreased in high SBM treatments, which is produced by macrophages and intestinal epithelial cells and is also one of key cytokines related to pro-inflammation [32]. The increased pro-inflammatory cytokines in intestinal mucosa might indicate a negative influence on the health of animals [33]. Interluekin-8, a pro-inflammatory cytokine, serves as immunoregulatory molecules that trigger immune cell activation, leading to inflammation [34]. The IL-8 is under transcriptional regulation by mitogen-activating protein kinase (MAPK), and it plays a pivotal role in neutrophil chemotaxis towards infection sites, thereby contributing to the induction of inflammation [35]. The F18+ *E. coli* challenge decreased IL-8 level in jejunal mucosa, which was different from previous studies [20,36]. However, Wong et al [37] found that certain inflammatory cytokines decreased and anti-inflammatory cytokines increased after 21 d *E. coli* challenge. In this study, we did not find significant impairments of intestinal health parameters between challenged and unchallenged groups, which might be explained by different F18+ *E. coli* dosages and types with previous study [26] or nursery pigs were in a recovery period and tried to maintain intestinal homeostasis.

In this study, the impaired growth performance was mainly attributed to reduced feed intake, and anorectic hormones are directly related to feed intake of pigs. Soy peptides have been indicated to reduce feed intake and increase metabolic rate [38]. A previous study suggested that the peptides (Leu-Pro-Tyr-Pro-Arg, Pro-Gly-Pro) from soybean glycinin showed anorectic activities [39]. In addition, several arginine-concentrated fragments in β-conglycinin have been indicated to bind intestinal cell components, especially the fragment from 51 to 63 of β-subunit, negatively affecting feed intake of rats via stimulating CCK in blood [40]. In the pig model, soybean protein hydrolysate, especially β-conglycinin hydrolysate stimulated CCK secretion and inhibited feed intake through calcium-sensing receptors [41]. In addition, researchers also found that nursery pigs preferred to eat a SBM based diet instead of a processed SBM diet [4]. The supplementation of SPC replacing animal protein supplements has been indicated to increase peptide tyrosine tyrosine which is an anorectic hormone limiting feed intake [23]. However, no difference in CCK was observed in this study. It is possible that SPC did not expose enough anorectic peptides in pig intestines, and that a 10% inclusion of SPC in the diets may not induce significant CCK secretion in nursery pigs.

Intestinal integrity and permeability are tightly related to the expression of specific tight junction proteins within the intestinal epithelial cells, including zona occludens 1 (ZO-1), claudin (CL), and OC [42]. The low SBM decreased the expression of OC in jejunal mucosa under F18+ *E. coli* challenge, which might be related to increased intestinal inflammation. The increased inflammation within the intestinal mucosa would result in enhanced oxidative stress and consequent damage to the intestinal epithelium [43]. Intestinal morphologies are highly related to nutrients absorption. Enhancements in villi height exhibit a positive correlation with the improved nutrient absorption capacity of villous enterocytes [44]. The damaged intestinal morphology was observed in pigs with diarrhea and was usually associated with a reduction in both digestive and absorptive functions [45,46]. In this study, the high SBM alleviated the damage of intestinal morphology under F18+ *E. coli* challenge compared to low SBM, which might be contributed to the increased inclusion of functional compounds in high SBM.

In conclusion, the replacement of SBM by SPC in nursery diets did not affect intestinal health and growth performance of pigs under unchallenged conditions. However, the high SBM inclusion in nursery diets mitigates the adverse impacts of F18+ *E. coli* challenge on the growth performance of pigs compared to low SBM inclusion replaced by SPC. This improvement was mainly attributed to decreased intestinal inflammation and enhanced intestinal integrity.

## Figures and Tables

**Table 1:** Composition of anti-nutritional compounds and isoflavones in soybean meal (SBM) and soy protein concentrate (SPC) (as-fed basis)

Item	SBM	SPC
Anti-nutritional compound (%)		
Stachyose	4.00	0.75
Raffinose	1.2	0.4
Galactose	<0.1	0.9
Sucrose	6.0	-
Trypsin inhibitor (mg/g)	2.0	1.1
Glycinin (mg/g)	44.3^1)^	<0.1
β-Conglycinin (mg/g)	120.2^2)^	<0.1
Isoflavone (mg/kg )		
Genistein	1,147	52.6
Daidzein	808	57.8
Glycitein	161	15.7

1) The values were obtained from Kim et al. [47].

2) The values were obtained from Smith and Dilger [29].

**Table 2:** The composition of experimental diets (as-fed basis)

Item	Phase 1		Phase 2	
	
	Low SBM	High SBM	Low SBM	High SBM
Feedstuff (%)				
Corn (yellow dent)	38.56	35.78	47.67	44.91
Soybean meal (dehulled)	12.06	24.00	14.06	26.00
Soy protein concentrate	10.00	0.00	10.00	0.00
Corn DDGS	5.00	5.00	5.00	5.00
Fish meal	7.00	7.00	3.50	3.50
Poultry meal	3.50	3.50	2.00	2.00
Poultry fat	1.80	2.60	2.30	3.10
Whey permeate	20.00	20.00	13.00	13.00
L-Lys HCl	0.48	0.48	0.46	0.46
DL-Met	0.20	0.21	0.16	0.17
L-Trp	0.03	0.03	0.01	0.01
L-Thr	0.18	0.18	0.13	0.13
L-Val	0.01	0.04	0.00	0.02
L-His	0.03	0.03	0.00	0.00
Dicalcium phosphate	0.00	0.00	0.46	0.50
Limestone	0.75	0.75	0.85	0.80
Vitamin premix^1)^	0.03	0.03	0.03	0.03
Mineral premix^2)^	0.15	0.15	0.15	0.15
Salt	0.22	0.22	0.22	0.22
Calculated composition				
DM (%)	92.05	91.98	91.17	91.10
ME (kcal/kg)	3,407	3,409	3,406	3,409
CP (%)	23.84	23.77	22.05	21.99
SID Lys (%)	1.50	1.50	1.35	1.35
SID M+C (%)	0.82	0.82	0.74	0.74
SID Trp (%)	0.25	0.25	0.22	0.22
SID Thr (%)	0.88	0.88	0.79	0.79
SID Val (%)	0.95	0.95	0.88	0.86
SID Ile (%)	0.84	0.80	0.80	0.76
SID Leu (%)	1.61	1.57	1.58	1.54
SID Phe (%)	0.92	0.88	0.90	0.86
SID His (%)	0.52	0.52	0.48	0.48
Lactose (%)	17.00	17.00	11.05	11.05
Ca (%)	0.85	0.85	0.80	0.80
STTD P (%)	0.48	0.48	0.40	0.40
Total P (%)	0.71	0.71	0.64	0.64
Analyzed composition, as-is				
DM (%)	90.21	90.02	89.50	89.40
Crude protein (%)	23.53	23.49	22.26	22.18

SBM, soybean meal; DDGS, distiller’s dried grains with soluble; Lys, lysine; Met, methionine; Val, valine; His, histidine; DM, dry matter; ME, metabolizable energy; CP, crude protein; SID, standardized ileal digestible; M+C, methionine+cysteine; Trp, tryptophan; Thr, threonine; Ile, isoleucine; Leu, leucine; Phe, phenylalanine; His, histidine; STTD P, standardized total tract digestible phosphorus.

1) The vitamin premix provided per kilogram of complete diet: 6,614 IU of vitamin A as vitamin A acetate, 992 IU of vitamin D_3_, 19.8 IU of vitamin E, 2.6 mg of vitamin K as menadione sodium bisulfate, 0.03 mg of vitamin B_12_, 4.6 mg of riboflavin, 18.5 mg of D-pantothenic acid as calcium pantothenate, 26.5 mg of niacin, and 0.07 mg of biotin.

2) The trace mineral premix provided per kilogram of complete diet: 30 mg of Mn as manganous oxide, 100 mg of Fe as ferrous sulfate, 100 mg of Zn as zinc sulfate, 15 mg of Cu as copper sulfate, 0.3 mg of I as ethylene diamine dihydroiodide, and 0.3 mg of Se as sodium selenite.

**Table 3:** Growth performance of nursery pigs fed diets with soybean meal replacing soy protein concentrate under F18+ *E. coli* challenge

Item	Unchallenged		Challenged^1)^		SEM	p-value		
		
	Low SBM^2)^	High SBM^2)^	Low SBM^2)^	High SBM^2)^		Challenge	SBM	Challenge×SBM
BW (kg)								
d 0	6.6	6.6	6.6	6.6	0.3	0.965	0.988	0.988
d 7	6.6	6.7	6.5	6.7	0.3	0.613	0.221	0.590
d 11	8.1^a^	7.9^a^	6.3^b^	7.5^a^	0.5	<0.001	0.105	0.018
d 18	11.2^a^	10.8^ab^	8.1^c^	9.5^b^	0.7	<0.001	0.230	0.045
d 25	15.0^a^	14.4^ab^	11.2^c^	13.1^b^	0.8	<0.001	0.270	0.053
ADG (g/d)								
d 0 to 7	−4	8	−19	13	22	0.746	0.130	0.500
d 7 to 11	280^a^	293^a^	−46^b^	123^c^	52	<0.001	0.028	0.058
d 11 to 18	430	409	240	277	40	<0.001	0.805	0.377
d 18 to 25	545	523	439	503	38	0.109	0.592	0.273
d 7 to 25	467	453	273	353	34	<0.001	0.270	0.112
d 0 to 25	334^a^	313^ab^	182^c^	258^b^	35	<0.001	0.269	0.054
ADFI (g/d)								
d 0 to 7	92	95	66	102	21	0.520	0.182	0.254
d 7 to 11	323	343	143	269	55	0.001	0.037	0.132
d 11 to 18	566^a^	559^a^	299^b^	429^c^	51	<0.001	0.103	0.069
d 18 to 25	740	788	532	652	48	0.001	0.088	0.461
d 7 to 25	579	600	356	484	45	<0.001	0.037	0.128
d 0 to 25	456^a^	463^a^	276^b^	387^a^	42	<0.001	0.050	0.078
G:F								
d 0 to 7	−0.26	−0.05	−0.59	−0.11	0.40	0.484	0.231	0.636
d 7 to 11	0.87	0.86	−0.52	−0.04	0.58	0.040	0.674	0.663
d 11 to 18	0.76	0.74	0.78	0.63	0.07	0.411	0.164	0.273
d 18 to 25	0.73	0.67	0.82	0.81	0.07	0.088	0.531	0.688
d 7 to 25	0.81	0.76	0.75	0.73	0.04	0.288	0.377	0.771
d 0 to 25	0.73	0.68	0.64	0.65	0.03	0.102	0.585	0.480

*E. coli*, *Escherichia coli*; SBM, soybean meal; SEM, standard error of the mean; BW, body weight; ADG, average daily gain; ADFI, average daily feed intake; G:F, gain to feed.

1) F18+ *E. coli* challenge on d 7 after weaning.

2) Low SBM, 12.06% and 14.06% soybean meal in phase 1 and 2; High SBM, 24% and 26% soybean meal in phases 1 and 2.

a–c Within a row, values with different superscripts are significantly different at p<0.05.

**Table 4:** Fecal score of nursery pigs fed diets with soybean meal replacing soy protein concentrate under F18+ *E. coli* challenge

Item	Unchallenged		Challenged^1)^		SEM	p-value		
		
	Low SBM^2)^	High SBM^2)^	Low SBM^2)^	High SBM^2)^		Challenge	SBM	Challenge×SBM
Pre-challenge								
d 0 to 7	4.0	3.9	3.8	3.7	0.2	0.322	0.726	0.880
Post-challenge								
d 7 to 11	3.5	3.6	4.4	4.4	0.2	<0.001	0.916	0.538
d 11 to 18	3.2	3.4	3.6	3.9	0.2	<0.001	0.128	0.696
d 18 to 25	3.1	3.2	3.2	3.2	0.1	0.766	0.385	0.451

*Escherichia coli*, *E. coli*; SBM, soybean meal; SEM, standard error of the mean.

1) F18+ *E. coli* challenge on d 7 after weaning.

2) Low SBM, 12.06% and 14.06% soybean meal in phase 1 and 2; High SBM, 24% and 26% soybean meal in phases 1 and 2.

**Table 5:** Immune and oxidative status, tight junction proteins, and inflammation gene expression of nursery pigs fed diets with soybean meal replacing soy protein concentrate under F18+ *E. coli* challenge

Item	Unchallenged		Challenged^1)^		SEM	p-value		
		
	Low SBM^2)^	High SBM	Low SBM	High SBM		Challenge	SBM	Challenge×SBM
Jejunal mucosa (/mg of protein)								
Protein carbonyl (nmoL)	1.70	1.74	1.93	1.61	0.23	0.823	0.548	0.429
MDA (nmoL)	0.24	0.23	0.29	0.26	0.07	0.421	0.709	0.885
TNF-α (pg)	10.53	9.08	11.41	6.60	1.25	0.488	0.008	0.149
IL-6 (pg)	12.60	16.76	12.30	11.51	3.24	0.394	0.604	0.448
IL-8 (ng)	0.67	0.76	0.56	0.48	0.07	0.005	0.994	0.207
IgA (μg)	3.45	4.83	4.00	3.35	0.76	0.412	0.519	0.077
Tight junction proteins								
ZO-1	1.08	1.15	0.97	1.05	0.11	0.250	0.420	0.948
OC	1.01	1.26	1.23	1.40	0.13	0.147	0.085	0.911
CL-1	1.06	1.10	0.83	1.07	0.16	0.438	0.399	0.546
Inflammation genes								
COX-2	1.04	1.11	0.97	0.91	0.15	0.353	0.987	0.658
iNOS	1.16	0.91	0.79	0.88	0.27	0.442	0.751	0.499
IL-1β	1.30	0.83	0.79	0.59	0.16	0.023	0.040	0.402
TGF-β	1.12	0.94	0.92	0.81	0.16	0.202	0.278	0.697
IL-10	1.05	1.06	0.98	0.90	0.21	0.459	0.822	0.774

*Escherichia coli*, *E. coli*; SBM, soybean meal; SEM, standard error of the mean; ZO-1, zonula occludens-1; OC, occludin; CL-1, claudin-1; COX-2, cyclooxygenase 2; iNOS, inducible nitric oxide synthases; IL-1β, interleukin 1β; TGF-β, transforming growth factor-beta; IL-10, interleukin 10.

1) F18+ *E. coli* challenge on d 7 after weaning.

2) Low SBM, 12.06% and 14.06% soybean meal in phase 1 and 2; High SBM, 24% and 26% soybean meal in phases 1 and 2.

**Table 6:** Intestinal morphology and cholecystokinin of nursery pigs fed diets with soybean meal replacing soy protein concentrate under F18+ *E. coli* challenge

Item	Unchallenged		Challenged^1)^		SEM	p-value		
		
	Low SBM^2)^	High SBM	Low SBM	High SBM		Challenge	SBM	Challenge×SBM
VH (μm)	401	433	413	453	25	0.507	0.144	0.866
CD (μm)	308^a^	298^a^	256^b^	291^a^	10	0.003	0.211	0.022
VH:CD	1.31	1.48	1.62	1.57	0.10	0.036	0.542	0.247
Ki-67 (%)	35.9	37.2	39.3	35.5	6	0.602	0.447	0.113
CCK (pg/mL)	3.17	3.06	3.47	3.42	0.61	0.596	0.892	0.962

*Escherichia coli*, *E. coli*; SBM, soybean meal; SEM, standard error of the mean; VH, villus height; CD, crypt depth; VH:CD, villus height to crypt depth ratio; Ki-67, enterocyte proliferation rate in the crypt; CCK, cholecystokinin.

1) F18+ *E. coli* challenge on d 7 after weaning.

2) Low SBM, 12.06% and 14.06% soybean meal in phase 1 and 2; High SBM, 24% and 26% soybean meal in phases 1 and 2.

a,b Within a row, values with different superscripts are significantly different at p<0.05.
